# Establishing and Maintaining Social Relationships During Significant Life Events: The Role of Age

**DOI:** 10.1093/geronb/gbae144

**Published:** 2024-08-23

**Authors:** Sonja Radjenovic, Fiona S Rupprecht, Jana Nikitin

**Affiliations:** Department of Developmental and Educational Psychology, University of Vienna, Vienna, Austria; Department of Developmental and Educational Psychology, University of Vienna, Vienna, Austria; Department of Developmental and Educational Psychology, University of Vienna, Vienna, Austria; (Psychological Sciences Section)

**Keywords:** Age differences, Establishing new social relationships, Life circumstances, Maintaining existing social relationships, Well-being

## Abstract

**Objectives:**

We investigate how adults of different ages experience changes in their social relationships during significant life events. Based on different goal priorities, younger adults may benefit more from establishing new social contacts during a significant life event, whereas older adults may benefit more from maintaining existing relationships.

**Methods:**

To test these hypotheses, we conducted multilevel modeling with a sample of *N* = 6,688 participants aged 18–90 years who reported at least 1 significant life event in the past 2 years.

**Results:**

Both establishing new social relationships and maintaining existing relationships during significant life events were associated with higher levels of well-being. As predicted, these associations were moderated by age. Establishing new social relationships was more strongly associated with mental health and life satisfaction in younger adults, whereas maintaining existing relationships was more strongly associated with subjective well-being, physical health, and loneliness in older adults.

**Discussion:**

These findings provide valuable insights into the role of age in the change and stability of social relationships during significant life events.

Significant life events are characterized by an interruption of the everyday routine and a high degree of personal meaning (Luhmann et al., 2021). Significant life events occur in a social environment and can change social relationships in different ways (Bidart & Lavenu, 2005). People may turn to close family and friends for comfort and support (McMahon et al., 2020), may lose existing social ties (Sander et al., 2017), or build new ones (Kalmijn, 2012). Although these changes may result from the life event itself (e.g., divorce typically leads to a loss of social contacts, while marriage often enables new social contacts; Kalmijn, 2012), they may also depend on individual factors.

One of these factors is age. A meta-analysis of changes in social networks during life events (Wrzus et al., 2013) showed that life events that occur at certain ages exhibit specific changes in the network composition: Life events that typically occur in young adulthood (e.g., marriage) are associated with an increase in the global social network (i.e., the entirety of social relationships of an individual including family, friends, neighbors, coworkers, etc.); life events that typically occur in older adulthood (e.g., widowhood) are associated with a decrease in the global network but a maintenance of the personal or support network (i.e., closer relationships such as family and friends). In other words, younger adults typically tend to expand their social network during significant life events, whereas older adults typically tend to shrink their social network but maintain important relationships.

In the present study, we go beyond the description of age-related changes in social networks during life events and ask whether age influences how people *experience* these changes. Specifically, we investigate whether increasing social networks and maintaining social networks are differently associated with well-being in younger and older adults. We examine the main indicators of well-being that have been associated with social relationships, that is, positive and negative affect (English & Carstensen, 2014), mental (Diener & Seligman, 2002) and physical health (Umberson & Karas Montez, 2010), life satisfaction (Park & Kang, 2023), and loneliness (Heinrich & Gullone, 2006). These indicators are based on the biopsychosocial model that includes physical (physical health), psychological (mental health, subjective well-being, life satisfaction), and social aspects of well-being (loneliness; see Pressman et al., 2020).

We argue that across all age groups, both maintaining existing relationships and establishing new ones are positively associated with these indicators of well-being. However, maintenance might be more beneficial in older adulthood, whereas establishing might be more beneficial in younger adulthood. We test these hypotheses on a large age-heterogeneous sample of individuals who have experienced at least one significant life event in the two years prior to the survey.

## Social Relationships and Well-Being During Significant Life Events

At all ages, maintaining existing social relationships might be important for well-being when individuals face challenges associated with significant life events. Familiar and close relationships that provide ongoing support and understanding can contribute significantly to well-being at such times. Such relationships are usually associated with high levels of well-being through authentic, accepting, and supporting experiences (Venaglia & Lemay, 2017).

On the other hand, new relationships can offer new perspectives and opportunities for positive experiences. After the birth of a child, for example, contact with other parents can provide shared experiences and a supportive network. Or in times of loss, socializing with people who have gone through similar experiences can provide a sense of understanding and companionship.

Thus, both establishing and maintaining relationships should be positively associated with well-being during significant life events. Despite a lack of direct evidence in the current literature, we expect that this is the case during all life events, because relationships in general provide support and buffer stress during challenging times (Taylor, 2011).

## The Role of Age

Despite generally positive associations with well-being, establishing and maintaining relationships may be associated with well-being differently depending on age. The socioemotional selectivity theory (SST) emphasizes the role of perceived remaining lifetime for age-related changes in social goals and relationships (Carstensen et al., 1999). An unlimited future-time perspective in young adulthood leads to information-acquisition goals that are most successfully pursued in large social networks of close, peripheral, and new social contacts (Baltes & Carstensen, 1999). Accordingly, young adults are motivated to make new social contacts that provide them with information and resources for their future development (e.g., Fung & Carstensen, 2006). With increasingly limited future time in later life, the goals shift from information acquisition to emotion regulation. This shift may help to maintain high levels of well-being (Carstensen, 1992) and is accompanied by the maintenance of existing relationships with deeper emotional meaning (Nikitin et al., 2024). Studies have shown that older adults’ smaller, yet emotionally closer social networks are associated with high levels of satisfaction and low levels of loneliness (e.g., Lang et al., 1998). Similarly, older adults who maintain relationships with family and friends report better physical and mental health than those who focus on new relationships (Rook & Charles, 2017). Finally, older adults are often more satisfied with their existing relationships than younger adults, report more positive and fewer negative emotions (English & Carstensen, 2014), and invest more time in maintaining (close) relationships (Lang & Carstensen, 2002).

Apart from future-time perspective, age-related changes in perceived resources may drive the selective investment in relationships (Bühler & Nikitin, 2020). As people grow older, they experience a decline in developmental gains and an increase in developmental losses (Brandtstädter, 2009). Accordingly, they become more selective when investing time and energy (Hess, 2014). Applied to the social domain, studies have shown that older compared to younger adults are less motivated to seek social gains in the form of new and peripheral relationships (Nikitin & Freund, 2019), and that seeking new and peripheral relationships is less beneficial for older adults’ well-being (Nikitin & Freund, 2018).

Regarding life events, we hypothesize that older adults benefit more from maintaining their relationships during significant life events due to their emotion regulation goals and limited resources. Conversely, younger adults may benefit from new relationships during such periods because they have a desire for novelty and information and have the high resources typical of their life stage. To our knowledge, this is the first study to test this hypothesis.

## The Present Study

To summarize, we hypothesize that both establishing new social relationships (H1) and maintaining existing ones (H2) during significant life events are positively associated with the biopsychosocial indicators of well-being, that means, high levels of subjective well-being, physical health, mental health, and life satisfaction, and low levels of loneliness. In addition, establishing new social relationships should be more strongly associated with the indicators of well-being in younger adults (H3), whereas maintaining existing social relationships should be more strongly associated with the indicators of well-being in older adults (H4).

The data were collected online. Individuals who stated in a prescreening that they had experienced at least one of the 19 listed life events within the last 2 years were invited to participate in the study. To counteract the limitation of one measurement point, we included the time interval between the survey and the occurrence of the life event as a moderator in the analyses. The strength of the associations between establishing and maintaining relationships during the life event on the one hand and indicators of well-being at the time of the survey on the other should decrease the further back the event occurred (H5; see Nikitin et al., 2012; Suh et al., 1996). Finally, because extraversion changes with age (Wilt & Revelle, 2015) and individuals with high extraversion have more enjoyable contacts than individuals with low extraversion (Wilt & Revelle, 2015), we controlled for extraversion in the analyses.

## Method

### Participants and Procedure

The study comprises *N* = 6,688 participants (50.9% male, 48.9% female, and 0.2% nonbinary; age *M* = 48.18, standard deviation [*SD*] = 16.83, and age range 18–90 years). The age distribution of the participants was relatively even, with approximately 17% of participants in each decade, with the exception for those aged 70+, who accounted for 13.1% of the total sample. The participants were from Germany (78.9%), Austria (13.8%), and Switzerland (7%). A small proportion (0.2%) were German-speaking participants residing in other countries. The questionnaire did not inquire about racial or ethnical background of participants. The majority had completed vocational training (31.2%), followed by college or university education (28.8%), secondary school (16.7%), higher vocational school (15.4%), compulsory school (4.4%), and “other” education (3.5%). The Ethics Committee of the Department of Psychology of the University of Basel approved the data collecting (009-20-1). The recruitment of participants was conducted by Respondi, an ISO-accredited recruitment service. Participants gave informed consent at the beginning of the study. They received €3.60 for completing the 20- to 30-min questionnaire. Data collection took place between June 23 and July 8, 2020.

### Measures

Detailed information on Cronbach’s alpha, means, *SD*s, and correlations for all variables are reported in Table 1. The exact wording of the items can be found in Supplementary Table 1.

**Table 1.** Cronbach’s α, Means, *SD*s, and Correlations

Variable	Cronbach’s α	*M*	*SD*	1.	2.	3.	4.	5.	6.	7.	8.	9.
1. Age		48.18	16.83									
2. Temporal distance		34.06	9.04	0.03*								
3. Extraversion	0.80	4.41	1.36	0.08**	0.01							
4. Establishing new social relationships		1.78	2.27	−0.25**	−0.01	0.12**						
5. Maintaining existing social relationships		3.90	1.53	−0.01	0.03*	0.10**	0.19**					
6. Subjective well-being	0.87	4.63	1.12	0.18**	0.08**	0.31**	0.10**	0.20**				
7. Physical health		4.76	1.52	−0.27**	0.09**	0.14**	0.18**	0.16**	0.42**			
8. Mental health		4.87	1.56	0.05**	0.07**	0.26**	0.13**	0.19**	0.71**	0.51**		
9. Life satisfaction	0.91	4.58	1.45	0.03*	0.05**	0.28**	0.13**	0.21**	0.61**	0.46**	0.61**	
10. Loneliness	0.92	2.17	1.20	−0.12**	−0.03**	−0.34**	−0.06**	−0.18**	−0.56**	−0.27**	−0.53**	−0.51**

*Notes*: *SD* = standard deviation. *N* = 6,688.

*p < .05. **p < .01.

#### Significant life events

A list of 19 significant life events, such as death of a loved one, job change, or marriage, was read by the participants. Life events were adapted from Household, Income and Labour Dynamics in Australia (Wooden et al., 2002) and Swiss Household Panel (Voorpostel et al., 2016; see Supplementary Table 1 for the list of all life events and their frequency in the sample). Participants were asked to select the one life event that had the greatest impact on their lives in the 2 years prior to the study. Participants who selected the “other” category indicated a life event that was not covered by any of the events listed.

#### Establishing new social relationships

Participants answered the question “Did you make new social contacts during the event?” with the answers “Yes”/“No.” If they answered “Yes,” they were asked how many new social contacts they had made during the event, with responses ranging from 1 = “very few” to 7 = “very many.” The two items were combined into one variable with “No” responses being represented as the value 0.

#### Maintaining existing social relationships

Participants answered the question “Do you have more or less contact with your existing social contacts than before the event?” using a 7-point response scale, ranging from 1 = “less than before” to 7 = “more than before.”

#### Subjective well-being

The participants reported their subjective well-being in the last 3–4 weeks, indicating the frequency (1 = *never* to 7 = *very often*) of feelings such as “sleepy,” “alert,” “tense,” and “calm” (Multidimensional Mood Questionnaire, Steyer et al., 1997; for all items, see Supplementary Table 1). If applicable, the items were recoded before being combined into a single scale. Higher scores indicate higher subjective well-being.

#### Subjective physical and mental health

The participants rated their physical and mental health by answering two questions, one on physical health and one on mental health: “All in all, how would you rate your physical/mental health?” (Idler & Benyamini, 1997), with responses ranging from 1 = “very poor” to 7 = “excellent.”

#### Life satisfaction

We used the five items of the Satisfaction with Life Scale (Diener et al., 1985) to assess life satisfaction (1 = “not true at all” to 7 = “very much true”). The items were combined into one scale, with higher scores indicating higher life satisfaction.

#### Loneliness

We used the 10-item version of the University of California, Los Angeles Loneliness Scale (UCLA) (Russell et al., 1980) to assess loneliness (1 = “not true at all” to 7 = “very much true”). The items were recoded were appropriate and aggregated into a scale, with higher scores indicating higher levels of loneliness.

#### Temporal distance

Participants indicated at monthly intervals when the life event ended (“the event is still ongoing,” “less than a month ago,” “1 month ago,” “2 months ago,” … , “24 months ago,” “more than 24 months ago”; see Author Notes point 1 for further information). The variable was used to measure how much time had elapsed since the life event and it was mean-centered for the analyses.

#### Extraversion

Participants indicated the extent to which statements such as “I am rather reserved” apply to them (1 = “not true at all” to 7 = “very much true”; Rammstedt & John, 2005). The four items were recoded if needed and aggregated into a scale with higher scores indicating higher levels of extraversion.

### Data-Analytical Strategy

The analysis plan for this study was preregistered and can be accessed at the following link: https://osf.io/n9zqm. To examine the data, we employed multilevel modeling (MLM; see Author Notes point 2 for further information), wherein specific life events were considered at Level 2, with individuals nested within these life events at Level 1. This approach enabled us to distinguish the variance between and within the life events. At Level 1, the individual values of establishing and maintaining relationships were used as predictors of the individual indicators of well-being. We additionally compared models with and without random slopes to test whether the associations differed between the life events. Age and temporal distance were included as moderators and extraversion as a covariate at Level 1. At Level 2, the life-event-specific average values for establishing and maintaining relationships were included as predictors of the life-event-specific averages in the indicators of well-being. This procedure enabled us to account and control for differences between life events (e.g., if a life event was associated with a higher mean score on establishing new relationships, this could be related to a higher mean score on the indicators of well-being). All predictors, moderators, and covariates were grand-mean-centered. The statistical analyses were conducted using R version 4.2.3 (R Core Team, 2023) and employed the following packages: “nlme” (v. 3.1-162, Pinheiro et al., 2023), “misty” (v. 0.4.11, Yanagida, 2023), and “reghelper” (v. 1.1.1, Hughes & Beiner, 2022).

## Results

### Descriptive Analyses

The frequency of life events and the mean age in the life events are listed in Supplementary Table 2. Across all life events, less than half of participants (44.87%) reported having established new relationships during the significant life event, suggesting that the life events did not lead to a substantial increase in relationships for most of the individuals. Regarding maintenance, we found a moderate stability of existing relationships with a slight tendency toward less contact during the significant life event (a significant deviation from the scale midpoint, *t* = −5.38, *df* = 6,687, *p* < .001; see Table 1 for means and *SD*s). The positive correlation between establishing new and maintaining existing relationships (see Table 1) indicates that participants who established more relationships also had more contact with their existing relationships. There was no significant correlation between maintaining existing relationships and age. In contrast, establishing new relationships was negatively correlated with age.

The intraclass correlation coefficients (ICCs) indicated that 25% (establishing; ICC = 0.25) and 4% (maintaining; ICC = 0.04) of the variance in social relationships can be attributed to the specific life event and the remaining variance to the individual and error variance. As for the indicators of well-being, the ICCs ranged from 0.05 (subjective well-being) to 0.11 (physical health). Following Luo and Lai’s (2021) suggestion to apply MLM for ICCs above 0.05, we used MLM for the following analyses.

### Social Relationships and Indicators of Well-Being During a Significant Life Event (H1 and H2)

We hypothesized that establishing new and maintaining existing relationships during the life event would be positively associated with the indicators of well-being. At the individual level (Level 1), establishing new relationships was positively associated with physical health, mental health, and life satisfaction (see Table 2). The associations between establishing new relationships and subjective well-being and loneliness were statistically significant only in models not controlling for extraversion (see Supplementary Table 3). Thus, these associations might be explained by extraversion. Maintaining existing relationships was associated with all indicators of well-being in the predicted way, even when controlling for extraversion. At the level of the life events (Level 2), establishing new and maintaining existing relationships were only associated with the indicators of well-being in 2 out of 10 cases. The predictors explained between 18% and 49% of the variance at the life-event level and between 4% and 13% of the variance at the individual level. However, the variance at the life-event level was in a large part explained by extraversion and life-event-specific means.

**Table 2.** Multilevel Models of Fixed and Random Effects for Social Relationships (During Significant Life Events) on Well-Being Outcomes

Variable	Subjective well-being	Physical health	Mental health	Life satisfaction	Loneliness
Fixed effects	**4.628** (0.060)	**4.838** (0.115)	**4.845** (0.091)	**4.536** (0.103)	**2.225** (0.060)
Intercept	**4.624** (0.049)	**4.780** (0.086)	**4.824** (0.069)	**4.526** (0.083)	**2.214** (0.056)
Level 1 (individual)					
Extraversion	**0.235** (0.010)	**0.117** (0.013)	**0.271** (0.013)	**0.263** (0.012)	**−0.281**(0.010)
Establishing new social relationships	0.012 (0.007)	**0.044** (0.009)	**0.031** (0.009)	**0.040** (0.008)	−0.006(0.007)
Maintaining existing social relationships	**0.115** (0.009)	**0.101** (0.012)	**0.142** (0.012)	**0.143** (0.011)	**−0.113**(0.009)
Level 2 (life event)					
Establishing new social relationships	0.009 (0.044)	**0.164** (0.077)	0.059 (0.063)	−0.005(0.074)	0.036 (0.050)
Maintaining existing social relationships	0.278 (0.157)	0.544 (0.273)	0.439 (0.222)	**0.587** (0.263)	−0.155(0.179)
Random effects					
Intercept	0.041 (39%)	0.131 (49%)	0.082 (47%)	0.122 (40%)	0.054 (18%)
Residual	1.073 (12%)	1.909 (4%)	2.116 (9%)	1.744 (10%)	1.211 (13%)

*Notes*: Controlled for extraversion and life-event-specific social relationship means. Fixed effects: standard errors in parentheses. Random effects: ∆*R*^2^ in parentheses. Significant effects in bold (*p* < .05).

The inclusion of random slopes generally improved the fit of the models, suggesting that the strength of the associations between building and maintaining relationships and the indicators of well-being varied across life events (see Supplementary Table 4 and Supplementary Text 1). In summary, maintaining relationships had the strongest positive effects on well-being outcomes for the life events “own serious illness and injury,” “unemployment,” and “retirement,” all of which may constitute significant loss experiences. Establishing relationships had the strongest positive effects for the life events “marriage,” “retirement,” “job change,” and “relocation to another country,” all of which may constitute experiences with new social contexts. Establishing relationships was furthermore negatively associated with well-being for the life events “long stay in hospital/institution” and “serious illness and injury of partner,” that is, events in the medical domain.

### Age and Temporal Distance as Moderators (H3–H5)

We examined whether age and temporal distance moderated the associations between establishing and maintaining relationships and the indicators of well-being. The moderation models included both predictors and moderators simultaneously and were conducted independently for each indicator.

**Figure 1.**
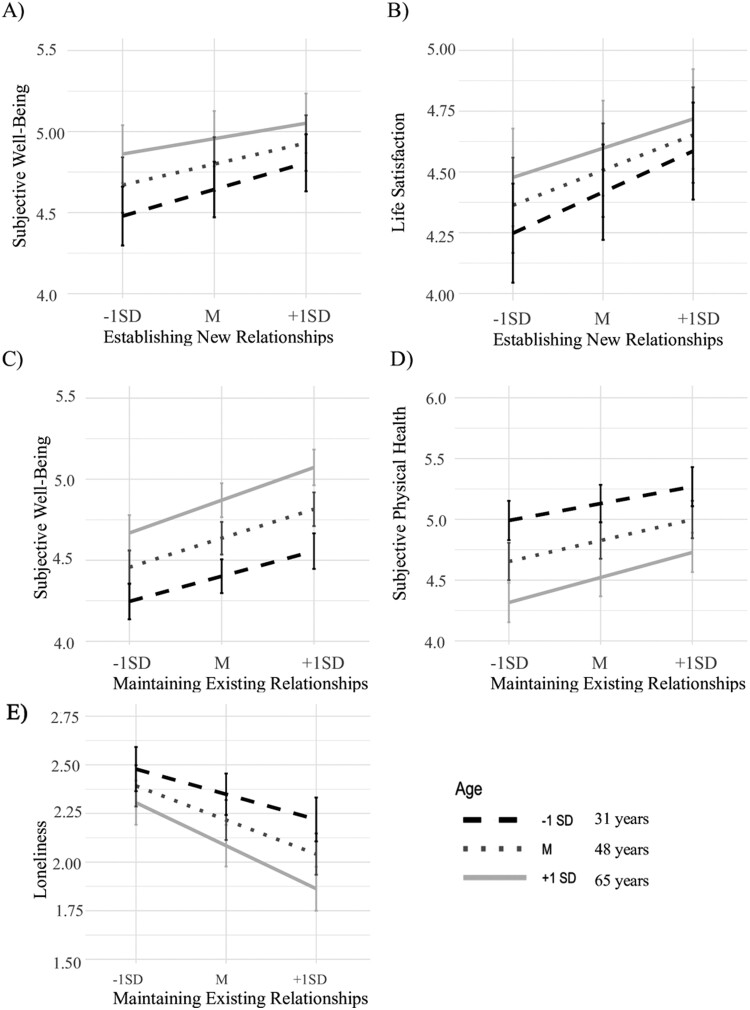
Significant multilevel age interactions between social relationships (establishing new ones: **A**, **B**; maintaining existing ones: **C**-**E**) and well-being outcomes. *SD* = standard deviation.

Supporting our hypotheses, age moderated the association between establishing new relationships and mental health and life satisfaction (see Table 3). As can be seen in Figure 1A and B, the associations were *weaker* with increasing age. In addition, age moderated the association between maintaining existing relationships and subjective well-being, physical health, and loneliness (see Table 3). The associations were *stronger* with increasing age (Figure 1C–E; see Author Notes point 3 for further information). Other interactions with age did not reach statistical significance.

**Table 3.** Multilevel Model of Fixed and Random Effects for Social Relationships (During Significant Life Events) on Well-Being Outcomes Moderated by Age and Temporal Distance

Variable	Subjective well-being	Physical health	Mental health	Life satisfaction	Loneliness
Fixed effects	**4.628** (0.060)	**4.838** (0.115)	**4.845** (0.091)	**4.536** (0.103)	**2.224** (0.060)
Intercept	**4.611** (0.053)	**4.794** (0.075)	**4.806** (0.079)	**4.515** (0.085)	**2.222** (0.059)
Level 1 (individual)					
Age	**0.014** (0.001)	**−0.018**(0.001)	**0.008** (0.001)	**0.005** (0.001)	**−0.007**(0.001)
Extraversion	**0.215** (0.009)	**0.139** (0.013)	**0.260** (0.013)	**0.256** (0.012)	**−0.270**(0.010)
Temporal distance	**0.010** (0.001)	**0.011** (0.002)	**0.013** (0.002)	**0.006** (0.002)	**−0.006**(0.002)
Establishing new social relationships	**0.023** (0.007)	**0.028** (0.009)	**0.034** (0.009)	**0.041** (0.009)	−0.011(0.007)
× Age	−0.000 (0.000)	−0.000 (0.000)	**−0.001**(0.000)	**−0.001**(0.000)	0.000 (0.000)
× Temp	**−0.001** (0.001)	**−0.002** (0.001)	−0.001(0.001)	**−0.002**(0.001)	0.000 (0.001)
Maintaining existing social relationships	**0.111** (0.008)	**0.105** (0.011)	**0.141** (0.012)	**0.143** (0.011)	**−0.112**(0.009)
× Age	**0.001** (0.000)	**0.001** (0.001)	0.001 (0.001)	−0.000(0.001)	**−0.002**(0.001)
× Temp	−0.001 (0.001)	−0.000 (0.001)	−0.002(0.001)	−0.002(0.001)	**0.002** (0.001)
Level 2 (life event)					
Establishing new social relationships	0.063 (0.048)	0.089 (0.068)	0.087 (0.072)	0.012 (0.076)	0.008 (0.053)
Maintaining existing social relationships	0.308 (0.170)	0.495 (0.240)	0.463 (0.253)	**0.598** (0.269)	−0.177(0.188)
Random effects					
Intercept	0.049 (27%)	0.099 (61%)	0.110 (29%)	0.128 (37%)	0.060 (9%)
Residual	1.018 (16%)	1.835 (7%)	2.085 (10%)	1.734 (11%)	1.192 (15%)

*Notes*: Controlled for extraversion and life-event-specific social relationship means. Fixed effects: standard errors in parentheses. Random effects: ∆*R*^2^ in parentheses. Significant effects in bold (*p* < .05).

We performed Johnson–Neyman intervals for significant age interactions to depict the exact range of significant age effects. The results showed significant effects for the entire age range (18–90 years) except for the interaction between establishing new relationships and mental health (significant age effects up to age 74) and establishing new relationships and life satisfaction (significant age effects up to age 86).

For temporal distance as a moderator, the effects reached significance for establishing new relationships and subjective well-being, physical health, and life satisfaction; and for maintaining existing relationships and loneliness. Here too, the results support our hypothesis that a greater time interval weakens the link between establishing relationships during the life event and the indicators of well-being during the survey. Results were contrary to the hypotheses for maintaining relationships and loneliness. Here, the association was stronger with time. Note that the moderation effects with temporal distance were generally stronger without the inclusion of extraversion as a control variable (see Supplementary Table 5).

In the moderation models, more variance was explained at the life-event level (between 27% and 61%, and 9% for loneliness; see Table 3). The proportion of variance explained at the individual level varied from 7% to 16%. When considering the additional explained variances through interactions, the age moderation explained considerable variance for physical health (26%). For all the other outcomes and for temporal distance as moderator, the additional explained variances were small (up to 5%).

Including random slopes for age improved the model fit for almost all models, whereas including random slopes for temporal distance improved the model fit for 5 out of 10 models (see Supplementary Table 7 and Supplementary Text 2).

## Discussion

The main aim of this study was to investigate the role of change and stability of relationships in well-being during significant life events and to explore whether age and temporal distance act as moderators of these associations. In summary, we found that (1) both establishing new and maintaining existing relationships was beneficial for various biopsychosocial indicators of well-being. Maintaining existing relationship showed hereby more robust associations with these indicators than establishing new relationships (which were more susceptible to the effects of extraversion). (2) Well-being was more strongly associated with differences in establishing and maintaining relationships *within* life events than by variations between individuals experiencing different life events. (3) Establishing relationships was more beneficial to the well-being of younger adults, while maintaining relationships was more beneficial to the well-being of older adults. (4) Time weakened the associations for some indicators of well-being.

### Social Relationships and Biopsychosocial Indicators of Well-Being

The results of the study are consistent with prior research that emphasizes the important role of social relationships for subjective well-being, health, life satisfaction, and loneliness, both in general (e.g., Diener & Seligman, 2002; English & Carstensen, 2014; Park & Kang, 2023) and during significant life events (Sippel et al., 2015). In general, maintaining existing relationships during significant life events had the stronger and more robust associations with the indicators of well-being compared to establishing new relationships. This emphasizes the great value of familiar and possibly also close relationships during times of major change.

Effects of establishing new social relationships appeared less robust. When controlling for extraversion, establishing new relationships remained a significant predictor of life satisfaction and health, but not for subjective well-being and loneliness. These results can be interpreted as a differential role of extraversion for more affective (subjective well-being and loneliness) versus cognitive (life satisfaction) and health-related outcomes (Schimmack et al., 2008) of social relationships. In any case, the role of extraversion should be considered when examining the impact of establishing new relationships on well-being during significant life events (see Nikitin & Freund, 2017, for a similar conclusion).

### The Role of Age

Our hypotheses that age moderates the relations between social relationships and the indicators of well-being were largely supported. These findings are consistent with both SST (Carstensen et al., 1999) and self-regulatory theories of development (Brandtstädter, 2009) and with related studies showing that older adults are more motivated by loss avoidance and therefore experience greater well-being from maintaining existing relationships, while younger adults are more motivated by gains and experience greater well-being from new relationships (Nikitin et al., 2012). To our knowledge, this is the first study to replicate these findings in the context of significant life events.

While we did not explicitly examine the nature of the existing relationships—whether they were close or peripheral—the convoy model of social relationships (Antonucci et al., 2014) supports the argument that the prioritization of existing relationships likely involves close and meaningful connections. The convoy model assumes that peripheral relationships usually dissolve first in older age, while social ties to close family and friends remain intact. Thus, one possible explanation of our findings could be that relationship closeness is a key factor for older adults’ well-being in times of change. However, future studies are needed to test the role of relationship closeness.

Although we found the hypothesized age moderations, they were not consistent across all indicators of well-being. Specifically, maintaining existing relationships was a stronger predictor of subjective well-being, physical health, and loneliness for older adults (and predicted mental health and life satisfaction irrespectively of age), whereas establishing new relationships was a stronger predictor of mental health and life satisfaction for younger adults (and predicted subjective well-being and physical health irrespectively of age). As these results were not predicted, we can only speculate about their meaning. For example, they could be interpreted in terms of different reactivity of younger and older adults to social changes during major life events or—in the case of reverse causality—as different resources needed at younger and older ages to build and maintain relationships. However, they could also simply be an expression of chance. Thus, we will have to wait for future studies before we can draw any conclusions. In the meantime, we can conclude that both establishing and maintaining relationships are important for well-being, with establishing being slightly more important in younger adulthood and maintaining in older adulthood.

### The Role of Temporal Distance

We expected that people become accustomed to the life event with increasing time distance and that the life event, and the social behavior tied to it would have less significance for the indicators of well-being of individuals with increasing temporal distance (Nikitin et al., 2012; Suh et al., 1996). Our results largely supported this hypothesis for the associations between establishing new relationships and indicators of well-being. The moderating effect of temporal distance was less consistent in the associations between maintaining existing relationships and indicators of well-being. One plausible interpretation is that the positive consequences of establishing new relationships may be rather immediate, whereas maintaining existing relationships (or a failure to do so) may come with more long-term consequences. In addition, we found that the moderating effect of temporal distance was influenced by extraversion (see Supplementary Table 5). It has been suggested that personality has a stronger influence on well-being when people are confronted with new situations, as is typical with significant life events (e.g., Caspi & Moffit, 1993). Highly extraverted individuals may turn to and benefit from supportive relationships from the start, while individuals with low extraversion need more time (Nikitin & Freund, 2015). In the end, however, the relationships of all individuals stabilize, and extraversion no longer plays a special role. Future research is needed to verify this argument.

### Life-Event-Specific Effects

It is crucial to recognize that certain life events bring with them different circumstances, commonly referred to as life-event-specific effects. This is reflected in the finding that the inclusion of random slopes in the models resulted in a better fit for all five indicators of well-being. In other words, the strength of the associations between maintaining existing and building new relationships and well-being depended to some extent on the context and challenges of the life event in question. Supplementary analyses suggest that maintaining relationships is particularly beneficial when it comes to life events that represent losses and challenges to existing social networks (e.g., unemployment, retirement, and own serious illness or injury), while establishing new relationships is particularly beneficial when it comes to life events that are associated with new social contexts (e.g., moving to another country, marriage, changing jobs). Going one step further, forming new relationships was even found to be unfavorable in life events related to the medical context (e.g., long hospital/institutional stay, serious illness or injury of a partner). In a medical context, making new social contacts with other patients, their families and healthcare professionals could be a sign of a debilitating and complex illness and a high need for support. Interestingly, when random slopes were considered, the positive associations with the indicators of well-being for the life event “retirement” emphasize the importance of both maintaining existing relationships and building new ones during this life event. This is in line with previous findings that some people benefit more strongly from existing social networks during retirement, while others use retirement as an opportunity to explore and build new relationships (Haslam et al., 2019).

It should be noted that the association between establishing new relationships and physical health was more consistent across life events (i.e., fewer random slopes) than for other indicators of well-being. The more robust association may suggest that optimal physical health plays a key role as a precursor to social relationships, rather than being a life-event-specific outcome. Individuals in good physical health are more likely to actively participate in social activities, which facilitates the establishment of new social interactions across a range of life circumstances (Domènech‐Abella et al., 2021).

Despite these life-event-specific effects, establishing and maintaining relationships explained a greater proportion of the variance in the indicators of well-being at the individual level than at the life-event level (except for loneliness). This finding supports the argument that it is not the specific life event that influences well-being, but individual factors (such as age) that shape the experience of life events. Future studies might therefore pay more attention to the mechanisms that describe well-being across life events rather than studying specific life events in isolation.

### Limitations and Implications for Future Research

The strengths of the current study lie in the large sample, the broad spectrum of significant life events, and the comprehensive consideration of various biopsychosocial indicators of well-being. However, some limitations should be noted.

Although the use of temporal distance as a moderator increases the validity of the cross-sectional results, it was not possible to test causality with our design. Longitudinal studies are needed to unravel the underlying mechanisms of the relationships found. In addition, some participants reported that the life event occurred more than 2 years ago, but the precise duration beyond 2 years remains unspecified. Consequently, the findings related to temporal distance should be interpreted with caution. In general, the retrospective assessment of life events carries the risk of recall biases influencing participants’ responses.

We did not include analyses on how the participants subjectively experienced the life events (e.g., their valence) or whether the findings differed for relationships of different quality. Relatedly, it would be important to consider cultural diversity in the role of social relationships for the individuals. For example, in countries with less welfare, where support is more dependent on informal relationships, the importance of maintaining existing social relationships during significant life events may be greater than in more developed countries (Torres & Casey, 2017).

Similarly, today’s older adults, who may have grown up in less secure economic and political circumstances, may place more value on maintaining their social networks than today’s younger adults who have been socialized in rapidly changing sociocultural contexts (Bühler & Nikitin, 2020). Therefore, the results of this study may not be generalizable to other cultures and age effects could be partly due to cohort differences.

In addition, societal ageism, which differs across countries (North & Fiske, 2015), may have an impact on how successful and beneficial establishing new social relationships can be for older adults. It is conceivable that in societies with strongly negative attitudes toward older adults, older adults may have fewer positive opportunities to form new social relationships. It would be an interesting topic for future research to investigate whether the benefits of establishing new social relationships for older adults differ depending on societal ageism.

Finally, the large number of analyses performed increases the risk of random significant results. We decided against the use of alpha-level corrections because they reduce statistical power and increase the risk of type II errors (Nakagawa, 2004). In addition, they disproportionately emphasize significance levels over effect sizes and explained variance, indicators that provide more meaningful information.

## Conclusion

Despite these limitations, our findings suggest that both establishing and maintaining relationships during significant life events are related to well-being, with establishing being more beneficial in younger adults and maintaining being more beneficial in older adults. Age is therefore an important variable when it comes to social change and stability during challenging times. These associations are dynamic and partly diminish over time. They also differ from event to event. Despite some heterogeneity, establishing and maintaining relationships are important predictors of well-being across life events and throughout adulthood.

## Supplementary Material

Supplementary data are available at *The Journals of Gerontology, Series B: Psychological Sciences and Social Sciences* online.

gbae144_suppl_Supplementary

## Data Availability

The study was preregistered at OSF (https://osf.io/n9zqm). Data and study materials are available from the first author upon request.

## Author Notes

1. Although participants read the instruction to only participate in the study if the life event occurred within the last 2 years, there were still 481 participants who selected the “more than 24 months ago” option. As time distance was not the main focus of the study, we decided not to exclude these participants from the sample.

2. We used multilevel analyses instead of the initially preregistered structural equation approach because multilevel analyses reflect the nested structure of the data (individuals within events) and thus provide a more detailed and comprehensive examination of the mean values specific to each life event.

3. We conducted exploratory analyses with gender as a moderator in two-way interactions with establishing and maintaining social relationships and in three-way interactions with age. Two significant gender interactions with social relationship variables indicated that compared to men, women experienced a higher subjective well-being and lower loneliness associated with maintaining existing relationships. These results imply that women may derive more benefits from maintaining relationships in terms of subjective well-being and loneliness reduction compared to men (see Supplementary Table 6). No other analyses led to systematic changes in the results.
