# Supplementary material for: Establishing and Maintaining Social Relationships During Significant Life Events: The Role of Age
Source: J Gerontol B Psychol Sci Soc Sci. 2024 Aug 23;79(11):gbae144. doi: 10.1093/geronb/gbae144 (PMC11475625; doi:10.1093/geronb/gbae144)
Supplement: gbae144_suppl_Supplementary [file gbae144_suppl_supplementary.docx]

***The Journals of Gerontology, Series B: Psychological Sciences and Social Sciences* Supplementary Material: Radjenovic et al. Establishing and Maintaining Social Relationships During Significant Life Events: The Role of Age.**

Supplementary Table 1. *German and English Wording of the Items*

|  | German Item | English Translation | Subscale |
| --- | --- | --- | --- |
| 1 | Umzug in ein anderes Land | Relocation to another country | Life event |
| 2 | Umzug in eine andere Stadt/einen anderen Wohnort | Relocation to another city / place of residence | Life event |
| 3 | Wohnungswechsel am gleichen Wohnort | Change of residence in the same city | Life event |
| 4 | Längerer Aufenthalt im Ausland | Longer stay abroad | Life event |
| 5 | Eine neue romantische Beziehung eingegangen (von mindestens einem Monat Dauer) | Entered a new romantic relationship (of at least a month) | Life event |
| 6 | Heirat/ eingetragene Partnerschaft | Marriage / registered partnership | Life event |
| 7 | Trennung oder Scheidung | Separation or divorce | Life event |
| 8 | Eine wichtige soziale Beziehung beendet (ausser Trennung oder Scheidung) | Ended an important social relationship (other than separation or divorce) | Life event |
| 9 | Eigene schwerwiegende Krankheit oder Verletzung | Own serious illness or injury | Life event |
| 10 | Schwerwiegende Krankheit oder Verletzung des Partners/der Partnerin | Serious illness or injury of the partner | Life event |
| 11 | Schwerwiegende Krankheit oder Verletzung eines nahen Familienmitgliedes oder Freundes/Freundin | Serious illness or injury of a close family member or a friend | Life event |
| 12 | Tod des Partners/der Partnerin | Loss of the partner | Life event |
| 13 | Tod eines nahen Familienmitgliedes oder Freundes/Freundin | Loss of a close family member or a friend | Life event |
| 14 | Längerer Aufenthalt im Spital/Psychiatrie/Gefängnis oder vergleichbarer Institution | Long stay in hospital / psychiatry / prison or comparable institution | Life event |
| 15 | Beförderung im Job | Job promotion | Life event |
| 16 | Stellenwechsel | Job change | Life event |
| 17 | Arbeitslosigkeit | Unemployment | Life event |
| 18 | Verrentung | Retirement | Life event |
| 19 | Anderes Ereignis | Other life event | Life event |
| 20 | Geburt eines Kindes | Birth of a child | Life event |
|  | Die folgenden Fragen beziehen sich auf das von Ihnen gewählte Ereignis: X Geben Sie bitte an, wann das Ereignis beendet war. | The following questions relate to the event you have chosen: X  Please indicate when the event ended. |  |
| 1. | Das Ereignis dauert noch an. | The event is still ongoing. | Temporal distance |
| 2. | vor weniger als einem Monat | less than a month ago | Temporal distance |
| 3. | vor 1 Monat | a month ago | Temporal distance |
|  | … | .... | Temporal distance |
| 26. | vor 24 Monaten | 24 months ago | Temporal distance |
| 27. | vor mehr als 24 Monaten | more than 24 months ago | Temporal distance |
|  | Folgende Fragen beziehen sich auf neue soziale Kontakte im Zusammenhang mit dem Ereignis. | The following questions relate to new social contacts in connection with the event. |  |
| 1. | Haben Sie während der Zeit des Ereignisses neue soziale Kontakte geknüpft? | Did you make new social contacts during the time of the event? | Establishing New Social Relationships |
| 2. | Wie viele neue soziale Kontakte haben Sie während der Zeit des Ereignisses geknüpft? | The following questions relate to your existing social contacts in connection with the event. | Establishing New Social Relationships |
|  | Folgende Fragen beziehen sich auf Ihre bestehenden sozialen Kontakte im Zusammenhang mit dem Ereignis. | The following questions relate to your existing social contacts in connection with the event. |  |
|  | Haben Sie mehr oder weniger Kontakt mit Ihren bestehenden sozialen Kontakten als vor dem Ereignis? | Do you have more or less contact with your existing social contacts than before the event? | Maintaining Existing Social Relationships |
|  | Im Folgenden finden Sie eine Liste mit Wörtern, die verschiedene Stimmungen beschreiben. Bitte gehen Sie die Wörter der Liste nacheinander durch und klicken Sie jeweils die Antwort an, die am besten beschreibt, wie häufig Sie sich in den vergangenen Wochen so gefühlt haben. | Below is a list of words that describes different moods. Please go through the list of words one by one and click on the answer that best describes how often you have felt this way in the past few weeks. |  |
|  | Wie häufig haben Sie sich in den vergangenen 3-4 Wochen so gefühlt? | How often have you felt this way in the past 3-4 weeks? |  |
| 1. | schläfrig | sleepy | SWB |
| 2. | wohl | good | SWB |
| 3. | ausgeglichen | at ease | SWB |
| 4. | unglücklich | unhappy | SWB |
| 5. | wach | alert | SWB |
| 6. | unzufrieden | discontent | SWB |
| 7. | angespannt | tense | SWB |
| 8. | frisch | fresh | SWB |
| 9. | glücklich | happy | SWB |
| 10. | nervös | nervous | SWB |
| 11. | ermattet | exhausted | SWB |
| 12. | ruhig | calm | SWB |
|  | Alles in allem, wie schätzen Sie Ihre körperliche Gesundheit ein? | All in all, how would you rate your physical health? | Physical Health |
|  | Alles in allem, wie schätzen Sie Ihre seelische Gesundheit ein? | All in all, how would you rate your mental health? | Mental Health |
|  | Nachfolgend finden Sie fünf Aussagen, denen Sie zustimmen oder nicht zustimmen können. Nutzen Sie die Antwortskala, um das Ausmaß Ihrer Zustimmung anzugeben. | Below are five statements that you can agree or disagree with. Use the response scale to indicate your level of agreement. |  |
| 1. | In den meisten Bereichen entspricht mein Leben meinen Idealvorstellungen. | In most ways my life is close to my ideal. | Life Satisfaction |
| 2. | Meine Lebensbedingungen sind ausgezeichnet. | The conditions of my life are excellent. | Life Satisfaction |
| 3. | Ich bin mit meinem Leben zufrieden. | I am satisfied with my life. | Life Satisfaction |
| 4. | Bisher habe ich die wesentlichen Dinge erreicht, die ich mir für mein Leben wünsche. | So far, I have gotten the important things I want in life. | Life Satisfaction |
| 5. | Wenn ich mein Leben noch einmal leben könnte, würde ich kaum etwas ändern. | If I could live my life over, I would change almost nothing. | Life Satisfaction |
|  | Wie zufrieden sind Sie mit Ihren sozialen Kontakten im Allgemeinen? | How satisfied are you with your social contacts in general? |  |
| 1. | Ich habe niemanden, an den ich mich wenden kann. | I have no one to turn to. | Loneliness |
| 2. | Ich habe Menschen, die mir nahestehen. | I have people who are close to me. | Loneliness (R) |
| 3. | Ich fühle mich allein. | I feel lonely. | Loneliness |
| 4. | Ich kann mit anderen zusammen sein, wenn ich das will. | I can be with others when I want to. | Loneliness (R) |
| 5. | Ich fühle mich ausgeschlossen. | I feel left out. | Loneliness |
| 6. | Ich habe Menschen, die mich wirklich verstehen. | I have people who really understand me. | Loneliness (R) |
| 7. | Ich fühle mich von anderen isoliert. | I feel isolated from others. | Loneliness |
| 8. | Ich habe Menschen, mit denen ich sprechen kann. | I have people to talk to. | Loneliness (R) |
| 9. | Ich bin zu viel allein. | I'm alone too much. | Loneliness |
| 10. | Es gibt Menschen, an die ich mich wenden kann. | There are people I can turn to. | Loneliness (R) |
|  | Folgend sind einige Aussagen zur Selbstbeschreibung aufgelistet. Bitte geben Sie an, inwieweit diese Aussagen auf Sie persönlich zutreffen.  Ich... | Some self-description statements are listed below. Please indicate to what extent these statements apply to you personally.  I... |  |
| 1. | bin eher zurückhaltend, reserviert. | am rather reserved. | Extraversion (R) |
| 2. | bin begeisterungsfähig und kann andere leicht mitreißen. | generate a lot of enthusiasm. | Extraversion |
| 3. | bin eher der „stille Typ“, wortkarg. | tend to be quiet. | Extraversion (R) |
| 4. | gehe aus mir heraus, bin gesellig. | am outgoing, sociable. | Extraversion (R) |

*Note.* SWB = Subjective Well-Being.

Supplementary Table 2. *Frequencies of Individual Significant Life Events and Their Mean Age*

| Significant Life Event | *N* | *M* (age) |
| --- | --- | --- |
| Relocation to another country | 121 | 38.12 |
| Relocation to another city / place of residence | 466 | 39.20 |
| Change of residence in the same city | 281 | 45.23 |
| Longer stay abroad | 203 | 45.76 |
| Marriage / registered partnership | 233 | 44.23 |
| Entered a new romantic relationship (of at least a month) | 374 | 38.17 |
| Birth of a child | 585 | 39.21 |
| Separation or divorce | 302 | 43.36 |
| End of an important social relationship (other than separation or divorce) | 186 | 44.85 |
| Own serious illness or injury | 834 | 58.05 |
| Serious illness or injury of the partner | 315 | 59.52 |
| Serious illness or injury of a close family member or a friend | 390 | 49.78 |
| Loss of the partner | 136 | 64.81 |
| Loss of a close family member or a friend | 1,054 | 52.30 |
| Long stay in hospital / psychiatry / prison or comparable institution | 71 | 48.44 |
| Job promotion | 156 | 39.82 |
| Job change | 360 | 40.76 |
| Unemployment | 248 | 45.72 |
| Retirement | 253 | 62.89 |
| Other life event | 120 | 52.30 |

Supplementary Table 3. *Multilevel Models of Fixed and Random Effects for Social Relationships (During Significant Life Events) on Well-Being Outcomes without Extraversion as Control Variable.*

| Variable | Subjective Well-Being | Physical Health | Mental Health | Life Satisfaction | Loneliness |
| --- | --- | --- | --- | --- | --- |
| Fixed Effects | **4.628** (0.060) | **4.838** (0.115) | **4.845** (0.091) | **4.520** (0.090) | **2.225** (0.060) |
| Intercept | **4.619** (0.053) | **4.778** (0.088) | **4.817** (0.076) | **4.526** (0.083) | **2.220** (0.060) |
| Level 1 (Individual) | | | | | |
| Establishing New | **0.032** (0.007) | **0.055** (0.009) | **0.054** (0.010) | **0.063** (0.009) | **-0.030**(0.007) |
| Maintaining Exis. | **0.129** (0.009) | **0.108** (0.012) | **0.159** (0.012) | **0.159** (0.011) | **-0.131**(0.010) |
| Level 2 (Life event) | | | | | |
| Establishing New | -0.011(0.048) | 0.154 (0.079) | 0.035 (0.068) | -0.027(0.081) | 0.061 (0.054) |
| Maintaining Exis. | 0.314 (0.171) | 0.562 (0.279) | 0.480 (0.242) | **0.627** (0.286) | -0.198(0.193) |
| Random Effects |  |  |  |  |  |
| Intercept | 0.049 (27%) | 0.137 (46%) | 0.099 (37%) | 0.144 (29%) | 0.063 (5%) |
| Residual | 1.170 (4%) | 1.933 (2%) | 2.246 (3%) | 1.866 (4%) | 1.351 (3%) |

*Note.*  Establishing New = establishing new social relationships. Maintaining Exis. = maintaining existing social relationships. Controlled for social relationship means of all individual life events. Fixed effects: Standard errors in parentheses. Significant effects in bold (*p* <.05). Random effects: ∆R^2^ in parentheses.

Supplementary Table 4. *ANOVA Comparison of Models with and without Random Slopes with Extraversion as Control.*

| *Variable* | AIC | BIC | Log-Likelihood | *p* |
| --- | --- | --- | --- | --- |
| Subjective Well-Being | 19541.53 | 19595.99 | -9762.766 |  |
| Random Slope |  |  |  |  |
| Establishing New | 19531.92 | 19599.99 | -9755.958 | .001 |
| Maintaining Exis. | 19526.80 | 19594.87 | -9753.399 | <.001 |
|  |  |  |  |  |
| Physical Health | 23402.90 | 23457.36 | -11693.45 |  |
| Random Slope |  |  |  |  |
| Establishing New | 23406.90 | 23474.97 | -11693.45 | 1 |
| Maintaining Exis. | 23383.41 | 23451.48 | -11681.70 | <.001 |
|  |  |  |  |  |
| Mental Health | 24080.85 | 24135.31 | -12032.43 |  |
| Random Slope |  |  |  |  |
| Establishing New | 24076.05 | 24144.12 | -12028.03 | .01 |
| Maintaining Exis. | 24077.65 | 24145.72 | -12028.82 | .03 |
|  |  |  |  |  |
| Life Satisfaction | 22797.70 | 22852.16 | -11390.85 |  |
| Random Slope |  |  |  |  |
| Establishing New | 22794.07 | 22862.14 | -11387.04 | .02 |
| Maintaining Exis. | 22785.87 | 22853.94 | -11382.93 | <.001 |
|  |  |  |  |  |
| Loneliness | 20355.39 | 20409.85 | -10169.69 |  |
| Random Slope |  |  |  |  |
| Establishing New | 20349.73 | 20417.80 | -10164.86 | .008 |
| Maintaining Exis. | 20309.77 | 20377.84 | -10144.89 | <.001 |

*Note.* Establishing New = establishing new social relationships. Maintaining Exis. = maintaining existing social relationships. Controlled for extraversion and social relationship means of all individual life events. Degrees of freedom = 8, 10. AIC = Akakike information criterion. BIC = Bayesian information criterion. -- = no fit.

Supplementary Text 1:

*Comparison of Associations Between Social Relationships and Well-Being Outcomes in Individual Significant Life Events*

We conducted further analyses of individual life events, whenever multilevel results indicated the presence of random slopes. Starting with maintaining existing relationships as a predictor, associations with subjective well-being were strongest for the life events “unemployment” and “own serious illness or injury” (*b*s > 0.20, *p*s < .001), followed by “retirement”, “serious illness or injury of a close other”, “change of residence in the same city”, “end of important relationship”, “longer stay abroad”, “birth of a child”, “serious illness or injury of partner”, and “loss of a close other” (*b*s > 0.10, *p*s < .05). Associations between maintaining existing relationships and physical health were strongest for the life events “own serious illness or injury” and “separation or divorce” (*b*s > 0.20, *p*s < .001), followed by “retirement”, “unemployment” and “change of residence in the same city”, “serious illness or injury of close other”, and “serious illness or injury of partner” (*b*s > 0.10, *p*s < .05). Associations between maintaining existing relationships and mental health were strongest for the life events “unemployment” and “own serious illness or injury”, “retirement”, and “change of residence in the same city” (*b*s > 0.20, *p*s < .01), followed by “other life event”, “serious illness or injury of close other”, “loss of close other”, “end of important relationship”, “separation or divorce”, and “relocation to another city” (*b*s > 0.10, *p*s < .05). Associations between maintaining existing relationships and life satisfaction were strongest for the life events “own serious illness or injury”, and “loss of the partner” (*b*s > 0.20, *p*s < .01), followed by “end of important relationship”, “unemployment”, “retirement”, “change of residence in the same city”, “separation or divorce”, “relocation to another city”, “longer stay abroad”, “serious illness or injury of partner”, “job change”, and “loss of a loved one” (*b*s > 0.10, *p*s < .05). Lastly, associations between maintaining existing relationships and loneliness were strongest for the life events “loss of the partner” and “own serious illness or injury” (*b*s < -0.20, *p*s < .001), followed by “retirement”, “unemployment”, “end of important relationship”, “loss of a close other”, “serious illness or injury of partner”, “separation or divorce”, “other life event” and “serious illness or injury of close other” (*b*s < -0.10, *p*s < .05).

Associations between establishing new social relationships and subjective well-being were strongest and positive for the life events “retirement”, “marriage”, and “job change” (*b*s < 0.10, *p*s < .05) and were negative for the life events “long stay in hospital/institution”, “serious illness or injury of partner”, and “loss of a loved one” (*b*s < -0.08, *p*s < .05). Associations between establishing new social relationships and physical health were not life-event-specific, i.e., there was no random slope. Associations between establishing new social relationships and mental health were strongest and positive for the life events “relocation to another country”, “retirement”, “change of residence in the same city”, “marriage”, and “job change” (*b*s > 0.07, *p*s < .05) and were negative for the life events “long stay in hospital/institution” and “serious illness or injury of partner” (*b*s < -0.15, *p*s < .05). Associations between establishing new social relationships and life satisfaction were strongest and positive for the life events “other life event”, “marriage”, “retirement”, and “separation or divorce” (*b*s > 0.10, *p*s < .05) and were negative for the life events “long stay in hospital/institution” and “serious illness or injury of partner” (*b*s < -0.15, *p*s < .05). Lastly, associations between establishing new social relationships and loneliness were strongest and negative (i.e., as expected) for the life events “relocation to another country” and “retirement” (*b*s > -0.10, *p*s < .05) and they were positive for the life events “loss of a close other” and “new romantic relationship” (*b*s < 0.10, *p*s < .05).

Supplementary Table 5. *Multilevel Model of Fixed and Random Effects for Social Relationships (During Significant Life Events) on Well-Being Outcomes Moderated by Age and Temporal Distance Without Extraversion as Control Variable.*

| Variable | Subjective Well-Being | Physical Health | Mental Health | Life Satisfaction | Loneliness |
| --- | --- | --- | --- | --- | --- |
| Fixed Effects | **4.628** (0.060) | **4.838** (0.115) | **4.845** (0.091) | **4.536** (0.103) | **2.224** (0.060) |
| Intercept | **4.605** (0.057) | **4.790** (0.078) | **4.798** (0.085) | **4.507** (0.092) | **2.230** (0.063) |
| Level 1 (Individual) |  |  |  |  |  |
| Age | **0.016** (0.001) | **-0.016** (0.001) | **0.011** (0.001) | **0.007** (0.001) | **-0.011**(0.001) |
| Temporal Distance | **0.009** (0.002) | **0.010** (0.002) | **0.012** (0.002) | **0.006** (0.002) | **-0.005**(0.002) |
| Establishing New | **0.043** (0.007) | **0.040** (0.009) | **0.059** (0.010) | **0.065** (0.009) | **-0.036**(0.007) |
| Establishing New *Age | -0.000 (0.000) | -0.000 (0.000) | **-0.001**(0.000) | **-0.001**(0.000) | 0.001 (0.000) |
| Establishing New *Temp | **-0.002** (0.001) | **-0.002** (0.001) | -0.001(0.001) | -0.002(0.001) | 0.000 (0.001) |
| Maintaining Exis. | **0.123** (0.009) | **0.113** (0.011) | **0.156** (0.012) | **0.158** (0.011) | **-0.128**(0.010) |
| Maintaining Exis. *Age | **0.001** (0.001) | **0.001** (0.001) | 0.001 (0.001) | 0.000(0.001) | **-0.002**(0.001) |
| Maintaining Exis.*Temp | **-0.002** (0.001) | -0.001 (0.001) | **-0.003**(0.001) | **-0.003**(0.001) | **0.003** (0.001) |
| Level 2 (Life event) |  |  |  |  |  |
| Establishing New | 0.053 (0.052) | 0.083 (0.071) | 0.075 (0.077) | 0.001 (0.082) | 0.020 (0.057) |
| Maintaining Exis. | 0.346 (0.183) | 0.519 (0.250) | 0.508 (0.272) | **0.643** (0.291) | -0.225(0.201) |
| Random Effects |  |  |  |  |  |
| Intercept | 0.057 (15%) | 0.108 (58%) | 0.128 (18%) | 0.150 (26%) | 0.069 (--) |
| Residual | 1.099 (10%) | 1.868 (6%) | 2.202 (5%) | 1.848 (5%) | 1.319 (6%) |

*Note.* Establishing New = establishing new social relationships. Maintaining Exis. = maintaining existing social relationships. Controlled for social relationship means of all individual life events. Fixed effects: Standard errors in parentheses. Significant effects in bold (*p* <.05). Random effects: ∆R^2^ in parentheses.

Supplementary Table 6. *Multilevel Model of Fixed and Random Effects for Three-way Interactions of Social Relationships (During Significant Life Events), Age and Gender.*

| Variable | Subjective Well-Being | Physical Health | Mental Health | Life Satisfaction | Loneliness |
| --- | --- | --- | --- | --- | --- |
| Fixed Effects | **4.628** (0.060) | **4.838** (0.115) | **4.845** (0.091) | **4.536** (0.103) | **2.225** (0.060) |
| Intercept | **4.717** (0.055) | **4.811** (0.078) | **4.927** (0.081) | **4.533** (0.086) | **2.245** (0.061) |
| Level 1 (Individual) |  |  |  |  |  |
| Age | **0.014** (0.001) | **-0.017** (0.001) | **0.010** (0.002) | **0.008** (0.001) | **-0.011**(0.001) |
| Temporal Distance | **0.010** (0.001) | **0.011** (0.002) | **0.013** (0.002) | **0.006** (0.002) | **-0.005**(0.002) |
| Extraversion | **0.227** (0.009) | **0.141** (0.013) | **0.272** (0.013) | **0.258** (0.012) | **-0.268**(0.010) |
| Gender | **-0.216** (0.026) | -0.032 (0.035) | **-0.247**(0.037) | -0.036(0.034) | -0.048(0.028) |
| Age*Gender | -0.001 (0.002) | -0.002 (0.002) | -0.004(0.002) | **-0.008**(0.002) | **0.005** (0.002) |
| Establishing New | **0.022** (0.009) | **0.022** (0.012) | **0.037** (0.012) | **0.045** (0.011) | **-0.021**(0.009) |
| Establishing New*Age | -0.001 (0.000) | -0.000 (0.001) | **-0.001**(0.001) | -0.001(0.001) | 0.000 (0.001) |
| Establishing New*Gender | 0.001 (0.012) | 0.011 (0.016) | -0.006(0.017) | -0.008(0.015) | 0.018(0.013) |
| Establishing New *Age*Gender | 0.000 (0.001) | 0.000 (0.001) | 0.000 (0.001) | 0.000 (0.001) | 0.001 (0.001) |
| Establishing New*Temp | **-0.002** (0.001) | **-0.002** (0.001) | -0.001(0.001) | **-0.002**(0.001) | 0.000 (0.001) |
| Maintaining Exis. | **0.086** (0.012) | **0.107** (0.016) | **0.123** (0.017) | **0.128** (0.016) | **-0.080**(0.013) |
| Maintaining Exis.*Age | 0.001 (0.001) | 0.002 (0.001) | 0.001 (0.001) | -0.001(0.001) | **-0.002**(0.001) |
| Maintaining Exis.*Gender | **0.040** (0.017) | -0.004 (0.022) | 0.026 (0.024) | 0.027 (0.022) | **-0.062**(0.018) |
| Maintaining Exis.*Age*Gender | 0.001 (0.001) | -0.001 (0.001) | -0.000(0.001) | 0.002 (0.001) | 0.000 (0.001) |
| Maintaining Exis.*Temp | -0.002 (0.001) | -0.000 (0.001) | -0.002(0.001) | -0.002(0.001) | **0.002** (0.001) |
| Level 2 (Life event) |  |  |  |  |  |
| Establishing New | 0.058 (0.048) | 0.088 (0.069) | 0.081 (0.071) | 0.009 (0.075) | 0.009 (0.54) |
| Maintaining Exis. | 0.299 (0.170) | 0.493 (0.242) | 0.447 (0.251) | **0.607** (0.267) | -0.189(0.189) |
| Random Effects |  |  |  |  |  |
| Intercept | 0.049 (27%) | 0.101 (61%) | 0.108 (31%) | 0.125 (38%) | 0.061 (8%) |
| Residual | 1.006 (17%) | 1.836 (7%) | 2.071 (11%) | 1.730 (11%) | 1.188 (15%) |

*Note.* Establishing New = establishing new social relationships. Maintaining Exis. = maintaining existing social relationships. Controlled for extraversion and social relationship means of all individual life events. Male gender as reference category, non-binary omitted due to small number of participants (*N* = 16). Fixed effects: Standard errors in parentheses. Significant effects in bold (*p* <.05). Random effects: ∆R^2^ in parentheses.

Supplementary Table 7. *ANOVA Comparison of Models with and without Random Slopes with Extraversion as Control.*

| *Variable* | AIC | BIC | Log-Likelihood | *p* |
| --- | --- | --- | --- | --- |
| Subjective Well-Being | 19280.66 | 19375.95 | -9626.331 |  |
| Random Slope |  |  |  |  |
| Establishing New *Age | 19280.65 | 19437.20 | -9617.326 | .04 |
| Establishing New *Temp | -- | -- | -- | -- |
| Maintaining Exis. *Age | 19276.19 | 19432.74 | -9615.097 | .008 |
| Maintaining Exis. *Temp | -- | -- | -- | -- |
|  |  |  |  |  |
| Physical Health | 23212.87 | 23308.16 | -11592.43 |  |
| Random Slope |  |  |  |  |
| Establishing New *Age | 23209.79 | 23366.34 | -11581.90 | .01 |
| Establishing New *Temp | -- | -- | -- | -- |
| Maintaining Exis. *Age | -- | -- | -- | -- |
| Maintaining Exis. *Temp | -- | -- | -- | -- |
| Mental Health | 24064.27 | 24159.56 | -12018.13 |  |
| Random Slope |  |  |  |  |
| Establishing New *Age | 24048.91 | 24205.46 | -12001.45 | <.001 |
| Establishing New *Temp | 24026.95 | 24183.50 | -11990.48 | <.001 |
| Maintaining Exis. *Age | 24047.62 | 24204.17 | -12000.81 | <.001 |
| Maintaining Exis. *Temp | -- | -- | -- | -- |
|  |  |  |  |  |
| Life Satisfaction | 22838.72 | 22934.01 | -11405.36 |  |
| Random Slope |  |  |  |  |
| Establishing New *Age | 22812.18 | 22968.72 | -11383.09 | <.001 |
| Establishing New *Temp | 22828.30 | 22984.85 | -11391.15 | <.001 |
| Maintaining Exis. *Age | 22798.80 | 22955.35 | -11376.40 | <.001 |
| Maintaining Exis. *Temp | 22815.56 | 22972.10 | -11384.78 | <.001 |
| Loneliness | 20331.37 | 20426.66 | -10151.68 |  |
| Random Slope |  |  |  |  |
| Establishing New *Age | 20324.07 | 20480.62 | -10139.04 | .003 |
| Establishing New *Temp | 20323.16 | 20479.70 | -10138.58 | .002 |
| Maintaining Exis. *Age | 20286.02 | 20442.57 | -10120.01 | <.001 |
| Maintaining Exis. *Temp | 20272.68 | 20429.23 | -10113.34 | <.001 |

*Note.* Establishing New = Establishing New Social Relationships; Maintaining Exis. = Maintaining Existing Social Relationships. Controlled for Extraversion (BIG-5) and social relationship means of all individual life events. Degrees of freedom = 14, 23. AIC = Akakike information criterion. BIC = Bayesian information criterion. -- = no fit.

Supplementary Text 2:

*Comparison of Associations Between Social Relationships and Well-Being Outcomes with Age as a Moderator in Individual Significant Life Events*

Life event-specific interactions between social relationships and well-being outcomes were rarely significant, unsystematic, and sporadic. Most likely, our models were powered to test for the general presence of random slopes, but not the specificities with regard to certain life events.
